# Chemotherapy for the treatment of intracranial glioma in dogs

**DOI:** 10.3389/fvets.2023.1273122

**Published:** 2023-10-31

**Authors:** Roberto José-López

**Affiliations:** Hamilton Specialist Referrals – IVC Evidensia, High Wycombe, United Kingdom

**Keywords:** brain tumor, canine, central nervous system, chemotherapeutic agent, glial neoplasms, lomustine (CCNU), temozolomide

## Abstract

Gliomas are the second most common primary brain tumor in dogs and although they are associated with a poor prognosis, limited data are available relating to the efficacy of standard therapeutic options such as surgery, radiation and chemotherapy. Additionally, canine glioma is gaining relevance as a naturally occurring animal model that recapitulates human disease with fidelity. There is an intense comparative research drive to test new therapeutic approaches in dogs and assess if results translate efficiently into human clinical trials to improve the poor outcomes associated with the current standard-of-care. However, the paucity of data and controversy around most appropriate treatment for intracranial gliomas in dogs make comparisons among modalities troublesome. To further inform therapeutic decision-making, client discussion, and future studies evaluating treatment responses, the outcomes of 127 dogs with intracranial glioma, either presumed (*n* = 49) or histologically confirmed (*n* = 78), that received chemotherapy as leading or adjuvant treatment are reviewed here. This review highlights the status of current chemotherapeutic approaches to intracranial gliomas in dogs, most notably temozolomide and lomustine; areas of novel treatment currently in development, and difficulties to consensuate and compare different study observations. Finally, suggestions are made to facilitate evidence-based research in the field of canine glioma therapeutics.

## 1. Introduction

Gliomas are a group of primary central nervous system (CNS) tumors with histologic features of glial cells [predominantly astrocytes (astrocytomas), oligodendrocytes (oligodendrogliomas), or mixtures of glial cells] (1). They account for over 35% of primary brain tumors in adult dogs (2–4). They arise mainly in the cerebral hemispheres and have a predilection for middle- to old-age males and brachycephalic breeds belonging to the Bulldog’s phylogenetic clade (3–7). The most common histologic subtype is high-grade oligodendroglioma, representing almost 55% of all gliomas in dogs (5, 6, 8).

Recent advances in treatment of canine intracranial gliomas have been driven by a combination of improved access to advanced imaging and treatment modalities such as surgery, chemotherapy, and radiotherapy together with the recognition of canine glioma as a reliable translational model for human glioma (9–11). Intracranial gliomas in dogs are associated with a poor prognosis; however, meaningful statements regarding their natural biology and behavior in light of therapy are yet difficult to make due to the limitations of most studies: small case numbers, retrospective study design, and lack of histologic diagnosis including tumor typing and grading.

To date, it is unknown whether tumor type or grade influence prognosis in canine intracranial glioma. Diverging observations of recent studies most likely stem from their low case numbers, differences in data analysis, and lack of homogeneity of treatment modalities (6, 12). By contrast, recent evidence indicates that specific anticancer therapies, consisting of surgical debulking, radiotherapy, or chemotherapy (either alone or combined), provide a statistically significant survival benefit over palliative treatment alone in histologically confirmed gliomas (6). Furthermore, the appropriacy of chemotherapy for the treatment of intracranial gliomas in dogs has been debated lately (13, 14), since a systematic review of brain tumor treatment in dogs concluded that surgery and radiotherapy were similarly superior to chemotherapy at palliating disease (15).

Glioma is the most common of brain cancers in humans, representing 81% of all malignant brain tumors in adults (16). It is classified into four grades based on malignancy (1). Glioblastoma, a grade IV astrocytoma, is the most common subtype and the most aggressive (1, 17), with a median survival of 14.6 months even after surgery and adjuvant radiotherapy and chemotherapy, the best available standard-of-care (18, 19). This overall median survival resulted from the addition of the chemotherapeutic temozolomide to surgical debulking and radiotherapy, which increased survival by approximately 10 weeks with minimal additional toxicity (18, 19).

Very little has changed for the treatment of high-grade gliomas in humans since the above cited study on temozolomide in 2005 (18). Although temozolomide first came into clinical practice back in the 1980s, it is still the “go to” drug for glioblastoma (18–20). Other compounds have shown promise in preclinical studies but have failed to translate effectively into clinical trials (21–23). The reason for this is because animal models that have been used in the laboratory to select these compounds, have failed to accurately recapitulate human disease (11, 24).

**Figure 1:**
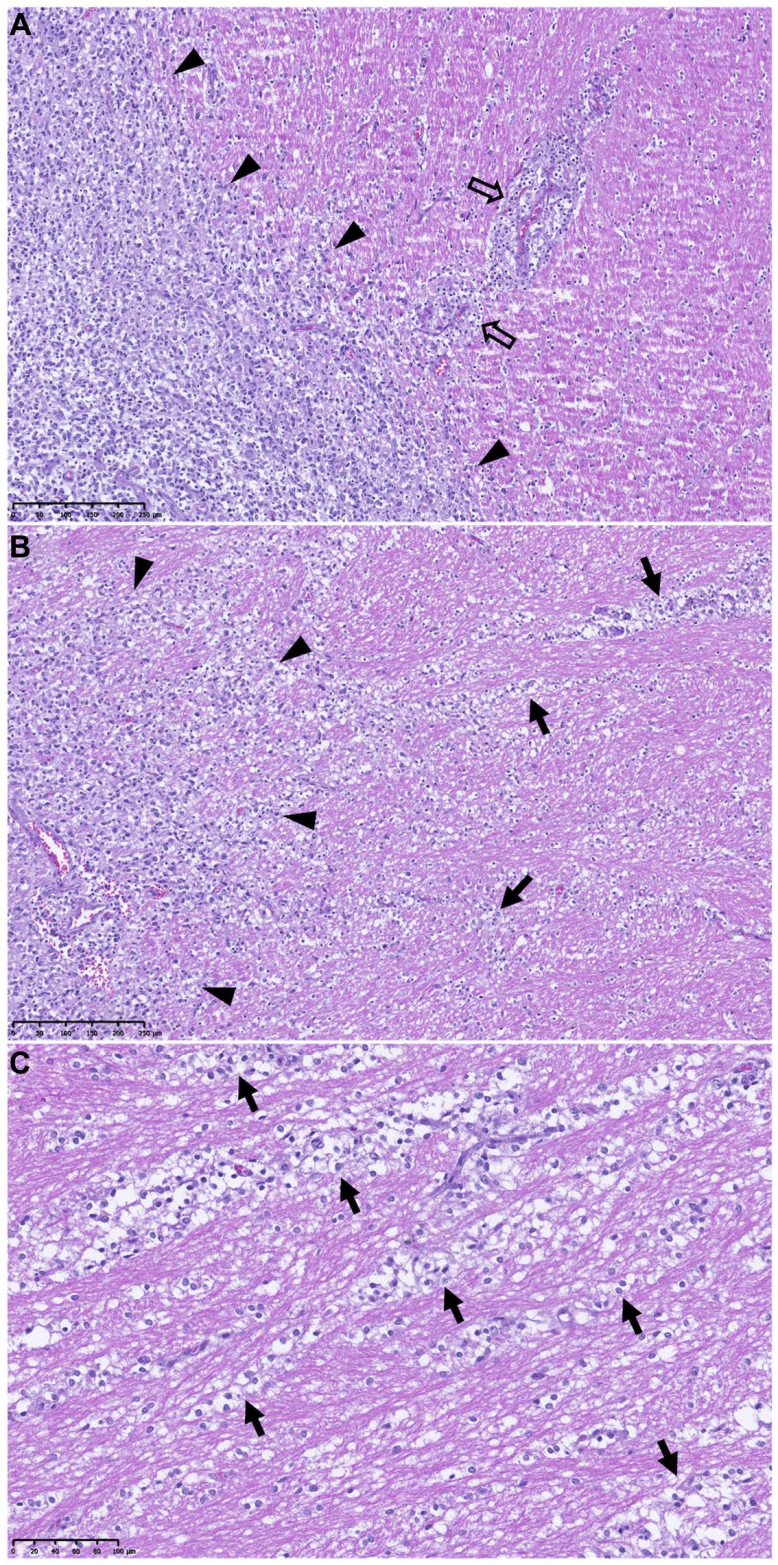
Secondary structures of Scherer demonstrating migration of glioma cells through normal brain structures. Periphery of a high-grade oligodendroglioma [outlined by black arrowheads on the left of pictures **(A, B)**] and adjacent neuropil in a dog. HE stains. **(A)** Perivascular aggregation of glioma cells (empty arrows). Scale bar = 250 μm. **(B)** Strings of tumor cells spreading along the surrounding white matter tracts (intrafascicular spread, black arrows). Scale bar = 250 μm. **(C)** Higher magnification of the tumor cells dissecting the white matter tracts beyond the tumor margins (black arrows). Scale bar = 100 μm.

**Figure 2:**
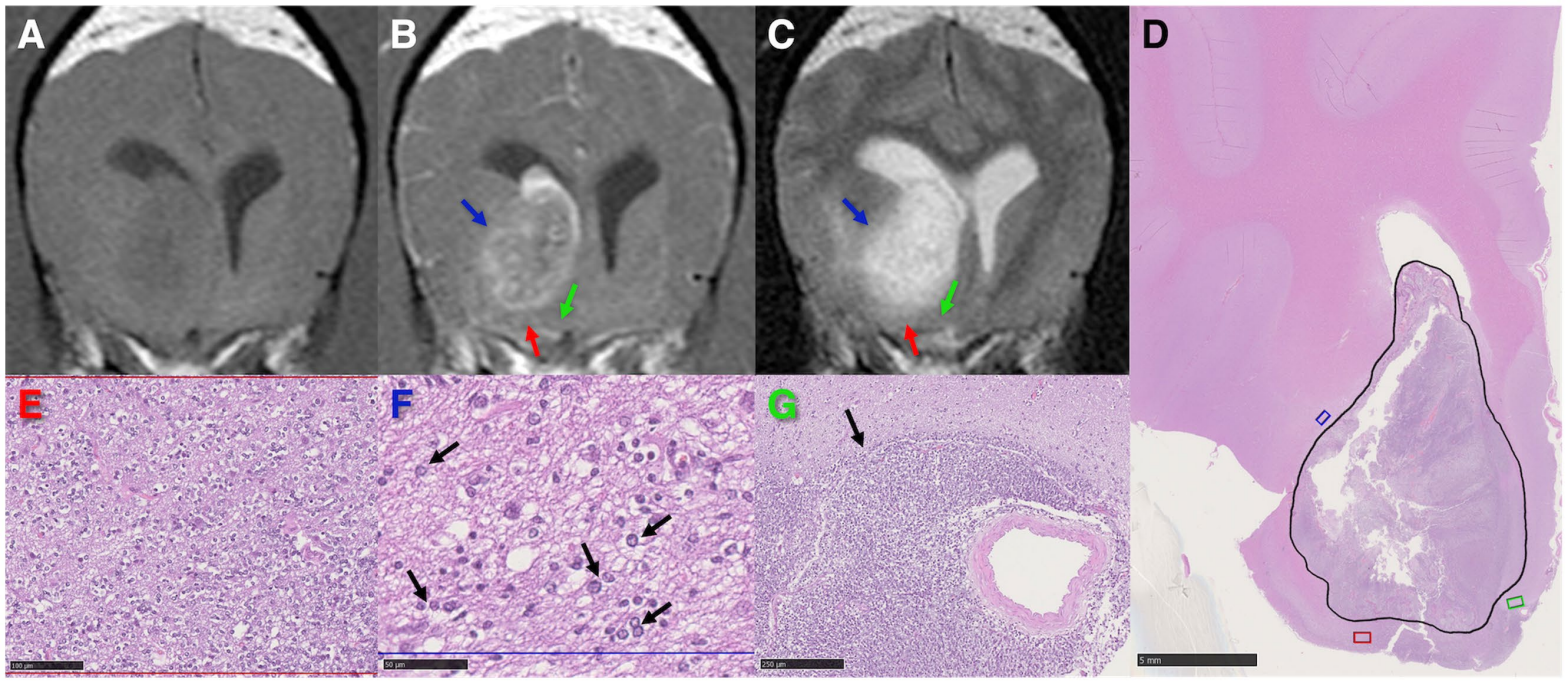
High-grade oligodendroglioma in the right caudate nucleus and internal capsule of a dog. **(A–C)** Transverse MR images demonstrating T1-weighted pre-contrast hypointensity **(A)**, post-contrast heterogeneous and partial ring enhancement **(B)**, and T2-weighted marked hyperintensity of the tumor **(C)**. The red and blue arrows highlight regions of peritumoral T2-weighted hyperintensity associated with edema, the green arrow points at an area of isointense signal to grey matter in all sequences. **(D)** Low magnification photomicrograph with the tumor area corresponding with the partial ring-enhancing lesion on **(B)** outlined in black. HE stain. Scale bar = 5 mm. The red, blue, and green areas correspond to the regions pointed out by the arrows of the same color in **(B, C)**. **(E)** Higher magnification of the red area in **(D)** revealing marked neoplastic cell invasion. HE stain. Scale bar = 100 μm. **(F)** Infiltrative tumor cells (black arrows) in a spongiotic neuropil (interstitial edema) in the blue rectangle in **(D)** coinciding with peritumoral T2-weighted hyperintensity **(C)** outside the partial ring-enhancing area **(B)**. HE stain. Scale bar = 50 μm. **(G)** Severe infiltration by tumor cells (black arrow) of the green area in **(D)**, apparently unaffected on MR images. HE stain. Scale bar = 250 μm.

There are multiple factors contributing to the poor outcomes associated with the current standard-of-care in human gliomas that canine gliomas share. Gliomas are intrinsic to the brain, the tumor cells invading cross normal anatomic boundaries, they spread along white matter tracts and frequently use blood vessels as guides (Figure 1) (5, 6, 25, 26). Infiltrative tumor cells can be histologically identified even centimeters away from the core lesion, outside the contrast enhancement area, both in regions of T2-weighted hyperintensity and regions apparently uninvolved (Figure 2) (27, 28). Diffuse invasion enables local recurrence after initial therapy as migrating glioma cells escape surgical resection, avoid the highest dose of radiotherapy, and involve regions with an intact blood–brain barrier (BBB), which diminishes chemotherapeutic availability.

Also, gliomas are thought to arise from cancer stem-like cells (CSCs), which have been identified both in human and canine tumors (29–34). Thus, a glioma is a heterogeneous mass of cells, containing differentiated cells but also CSCs and progenitor-like cells (29–35). Current radiotherapy and chemotherapy regimens only target the differentiated cells as CSCs appear to have an innate resistance (36–39), so differentiated cells disappear, the tumor bulk reduces, but the tumor recurs from the CSCs left behind. Besides, CSCs also exhibit a high motility contributing to glioma aggressive invasiveness (32).

## 2. Aims of the review

Novel treatment concepts based on the above biological insights are urgently needed, and canine glioma could be a suitable model for preclinical drug screening as well as clinical trials. In turn, this might inform on treatment and prognosis in canine patients. The natural biology of canine spontaneous glioma is poorly documented, as is response to conventional therapy such as surgery, radiation and chemotherapy. An overview of the current body of knowledge on responses of canine intracranial glioma to chemotherapy as leading or adjuvant treatment, both in clinical and experimental patients, is provided here. The objectives are to further inform the canine model and the assessment of new treatment strategies as well as to assist therapeutic decision-making and client discussion. Finally, suggestions are made to advance evidence-based research on canine glioma therapeutics.

## 3. Methods

Reports including chemotherapy as part of conventional or experimental treatments for dogs with intracranial glioma are scarce and frequently include cases with just a presumed clinical diagnosis based on magnetic resonance imaging (MRI) characteristics (4, 6, 40–43). In veterinary medicine, histologic diagnosis of glioma by means of surgical biopsy or necropsy is often hampered by the risk of the procedure in the case of biopsies, financial constraints, and owner decisions overall (6, 14, 44). Thus, Pubmed was used to search the literature for studies including both, dogs with histologically confirmed and presumed diagnosis of intracranial glioma. The following search terms were used: “dog AND chemotherapy AND glioma,” “oligodendroglioma AND chemotherapy AND dog,” “astrocytoma AND chemotherapy AND dog,” “canine AND glioma AND chemotherapeutic,” “dog AND glioma AND temozolomide,” “dog AND glioma AND lomustine,” and a total of 124 results were obtained. Of these, 17 reports on chemotherapy as part of the treatment for canine intracranial glioma with available outcome information were identified for review. *In vitro* studies, laboratorial research on murine models or healthy dogs, studies that failed to provide information on survival, and irrelevant articles due to description of other treatment modalities or repetition, were excluded.

## 4. *In vivo* treatments with chemotherapy as leading or adjuvant modality

Treatment modalities and their associated outcomes including median survival time (MST) when applicable, are outlined in Table 1. The MST for dogs with histologically confirmed intracranial glioma reported to receive palliative treatment consisting of corticosteroids, antiepileptic drugs, and analgesic medications as the sole therapeutic interventions is 25 days (range, 1.5–492 days; *n* = 23) (6, 45). This will be used as reference for comparison when discussing results of studies on chemotherapy. The range of MSTs for suspected cases of intracranial glioma treated palliatively is 37 days (*n* = 22) (45) to 94 days (*n* = 30) (46).

**Table 1:** Summary of published outcomes in dogs with histologically confirmed and presumed intracranial gliomas treated with chemotherapeutic agents alone or combined with other modalities.

Treatment modality	Histologically confirmed gliomas				Presumed gliomas				Comments
	References	n	Median survival time (days)	Range (days)	References	n	Median survival time (days)	Range (days)	
Palliative care	José-López et al. (6), Moirano et al. (45)	23	25	1.5–492	Moirano et al. (45)	22	37	2–181	Individual case outcome data not available in Dolera et al. (46).
					Dolera et al. (46)	30	94	Not applicable	
Oral lomustine	José-López et al. (6), Moirano et al. (45), Jeffery and Brearley (47)	5	39	18–120	Moirano et al. (45)	16	135	5–520	
Surgical resection and oral lomustine	José-López et al. (6), Jeffery and Brearley (47), Fulton and Steinberg (48)	6	165	60–720	No data				One case in Fulton and Steinberg (48) alive at the time of analysis (630 days).
Linear accelerator (linac) hypo-fractionated radiotherapy and oral lomustine	Hasegawa et al. (50) Nakamoto et al. (51)	2	633	356–910	No data				In dog surviving 910 days in Hasegawa et al. (50), lomustine started at day 120 as rescue due to progressive disease post-radiotherapy.
Stereotactic hypo-fractionated radiotherapy and oral lomustine	No data				Moirano et al. (52)	2	523	388–658	
Surgical resection, linac hypo-fractionated radiotherapy and oral lomustine	Jeffery and Brearley (47)	1	90	Not applicable	No data				
Oral temozolomide	José-López et al. (6)	1	190	Not applicable	No data				
Surgical resection and oral temozolomide	José-López et al. (6), Hidalgo Crespo et al. (53)	10	185	51–456	No data				One dog surviving 428 days in Hidalgo Crespo et al. (54) underwent repeat surgery and was euthanized 2 days after due to aspiration pneumonia.
Surgical resection, 1^st^ oral temozolomide then melphalan	Hidalgo Crespo et al. (53)	1	493	Not applicable	No data				Chemotherapy changed due to clinical deterioration suspected secondary to tumor progression.
Surgical resection, 1^st^ oral temozolomide then lomustine	Hidalgo Crespo et al. (53)	1	240	Not applicable	No data				Chemotherapy changed due to tumor progression confirmed on imaging.
Surgical resection x2, 1^st^ oral temozolomide then lomustine	Hidalgo Crespo et al. (53)	1	468	Not applicable	No data				Chemotherapy changed after reintervention for imaging confirmed tumor progression.
Surgical resection x2, 1^st^ oral temozolomide then toceranib phosphate	Hidalgo Crespo et al. (53)	1	241	Not applicable	No data				Chemotherapy changed after reintervention for imaging confirmed tumor progression.
Surgical resection x3, 1^st^ oral temozolomide then toceranib phosphate, then lomustine	Hidalgo Crespo et al. (53)	1	780	Not applicable	No data				Chemotherapy changed after each reintervention following respective confirmation of tumor progression on imaging.
Stereotactic hypo-fractionated radiotherapy and oral temozolomide	No data				Moirano et al. (52)	1	237	Not applicable	Lost to follow-up.
					Dolera et al. (46)	20	420	Not applicable	Individual case outcome data not available. Survival time calculated from the end of radiation treatment.
Surgical resection and intratumoral implantation of microcylinders conjugated with temozolomide	Hicks et al. (55)	4	37	9–120	No data				Survival described from treatment. One dog euthanized 9 days postoperatively for splenic hemangiosarcoma. Long-term outcome data not available for the rest, two cases alive at day 30 and 120, respectively, and one withdrawn from study on day 44.
Convection-enhanced delivery (CED) of temozolomide	Young et al. (56)	3	38	1–82	Young et al. (56)	6	89	44–722	Survival time calculated from temozolomide CED treatment. One patient alive at the time of data analysis (722 days).
Intravenous carmustine	Dimski and Cook (57)	1	213	Not applicable	No data				
Oral hydroxyurea and imatinib	Yun et al. (58)	1	1,155	Not applicable	No data				
Stereotactic hypo-fractionated radiotherapy and oral hydroxyurea	No data				Moirano et al. (52)	1	484	Not applicable	Lost to follow-up
Stereotactic hypo-fractionated radiotherapy and oral toceranib phosphate	No data				Moirano et al. (52)	1	257	Not applicable	
Subcutaneous cytarabine	José-López et al. (6)	4	22	2–84	No data				
CED of irinotecan hydrochloride	Dickinson et al. (59)	9	190	126–611	No data				Two dogs euthanized for unrelated reasons. Two cases alive at the time of data analysis (181 and 611 days, respectively).
CED of cetuximab following open biopsy or partial surgical resection	Freeman et al. (60)	8	248	103–903	No data				Survival time calculated from surgery.
Surgical resection, oral metronomic chlorambucil and lomustine	Bentley et al. (61)	8	257	64–860	No data				Individual case outcome data not available. Reported median survival time and range only for cancer-related deaths, three cases censored. Survival time measured from surgery.
Oral clomipramine	José-López et al. (6)	4	236	45–1,104	No data				
Targeted doxorubicin delivery via minicells	MacDiarmid et al. (62)	6	See “Comments” column		MacDiarmid et al. (62)	2	See “Comments” column		Survival specific to intracranial glioma cases unavailable. Median survival time for all included brain tumors 264 days (range, 49–973). Survival time definition not provided.

Outcomes reported for palliative care as sole therapeutic intervention are also included for comparison. When individual case outcome data could be extracted from reviewed articles, median survival times (MSTs) and ranges were calculated for all reported cases receiving the same therapeutic regimen. Survival times are reported in days from advanced imaging diagnosis unless specified otherwise in the “Comments” column.

### 4.1. Reported treatment regimens in canine patients with intracranial glioma

#### 4.1.1. Temozolomide

Temozolomide is an oral alkylating agent that produces DNA damage in a non-cell cycle specific manner by methylation of purine bases (O6-guanine; N7-guanine and N3-adenine) (20, 63). The exact mechanism of temozolomide-induced cell toxicity is enigmatic and involves autophagy, necrosis, senescence, and apoptosis (64). As stated above, it has become the standard-of-care for adjuvant therapy of high-grade gliomas in humans, but also, has demonstrated antitumor activity as single agent in the treatment of recurrent glioma (18–20, 65). However, its efficacy is determined by O6-methylguanine-DNA methyltransferase (MGMT; DNA repair enzyme) promoter methylation, present in only 40–50% of cases (66).

In dogs, *in vitro* studies have shown that glioma cell lines have responses to temozolomide similar to human glioma cell lines, with visible reduction in viable cell populations when tested alone and additive cytotoxic effect to irradiation when combined (67, 68). Preliminary studies profiling genome-wide methylation status of canine glioma suggest that hypermethylation patterns might be similar to those in human gliomas (69). In the clinical setting, temozolomide has been used as a therapeutic option for different tumor types in dogs and an oral dose of 60-100 mg/m^2^ once daily for 5 days on a 28-day cycle has traditionally been described (46, 54, 70, 71). The maximally tolerated dose of temozolomide for dogs after a single 5-day course has recently been established at 150 mg/m^2^ daily (72). Most frequently occurring adverse effects are hematologic and hepatic, followed by gastrointestinal, with the majority being self-resolving and of mild grade (46, 54, 70–72).

There is only one dog in the literature with detail on survival after temozolomide as the sole anticancer therapy for intracranial glioma in addition to palliative care with corticosteroids and phenobarbital (6). This case survived 190 days after MRI diagnosis and was confirmed as high-grade oligodendroglioma post-mortem.

Surgical debulking and temozolomide as single adjuvant chemotherapy (60–181.8 mg/m^2^ daily for 5 days on a 28-day cycle) has been reported in 10 dogs with confirmed intracranial glioma and a MST of 185 days (range, 51–456 days) from advanced imaging diagnosis (6, 53). Of these, one case had a second surgery after confirmed tumor progression and was euthanized 2 days later due to aspiration pneumonia, 428 days after imaging diagnosis. In the study by Hidalgo Crespo et al. temozolomide was the first-line adjuvant chemotherapy in five more intracranial gliomas but was changed to another chemotherapy agent when tumor progression was either suspected or confirmed (53). This included melphalan (0.15 mg/kg for 5 days every 3 weeks) in one case surviving 493 days; lomustine (70 mg/m^2^ every 3 weeks) in one dog that survived 240 days and another that also underwent repeated surgery and survived 468 days; toceranib phosphate (2.5–2.8 mg/kg three times weekly) in a dog that underwent surgical reintervention too surviving 241 days; and in one case with two relapses followed by subsequent repeated surgeries (survival time, 780 days), temozolomide was substituted by toceranib phosphate after the first relapse, and this was replaced by lomustine after the second relapse. Note that all the above dogs treated with temozolomide had a supratentorial glioma within the cerebral hemispheres except for one case receiving surgery too, that had it in the cerebellum and survived 120 days (6, 53).

A prospective study in dogs with presumed intracranial glioma evaluated frameless stereotactic radiotherapy alone and combined with temozolomide (46). Survival was calculated from the end of radiation therapy and no statistically significant advantage could be found in dogs irradiated in combination with temozolomide at low doses of 65 mg/m^2^ daily for 5 days monthly for six cycles (MST, 420 days; *n* = 20) when compared to dogs receiving radiotherapy alone (MST, 383 days; *n* = 22). Conversely, both modalities provided a significant survival benefit compared to palliative care (MST, 94 days; *n* = 30). Another study on frameless stereotactic radiotherapy for presumptive canine intracranial gliomas included one case receiving adjuvant temozolomide chemotherapy that was alive 237 days after MRI diagnosis, when it was lost to follow-up (52). All dogs reportedly treated with stereotactic radiotherapy and adjuvant temozolomide had supratentorial tumors, either hemispheric or diencephalic (six cases) (46, 52).

#### 4.1.2. Lomustine

Lomustine (1-(2-chloroethyl)-3-cyclohexyl-1-nitrosourea, CCNU) is a nitrosurea-based alkylating agent that undergoes hydrolysis to reactive metabolites and exerts its effects through alkylation, cross-linking and carbamylation (67, 73–75). After temozolomide, it probably is the most commonly used chemotherapeutic agent for human glioma treatment (75, 76).

*In vitro* studies have shown that treatment with lomustine results in decreased canine glioma cell viability similar to that reported for human glioma cell lines (67). In clinical canine patients, it is usually administered at doses of 30-90 mg/m^2^ orally every 3–8 weeks (45, 47–51, 77). Lomustine-associated toxicity is common but usually not life threatening (49). The adverse effects of lomustine in dogs are primarily attributable to bone marrow effects, followed by gastrointestinal, hepatic, and less commonly, renal (45, 49, 77). Maximal bone marrow effects (neutropenia) are generally seen 7 days after dosing (77). The doses cited above have been used to minimize the bone marrow effects. At doses of 100 mg/m^2^ excessive myelosuppression has been reported. Thrombocytopenia as a cumulative effect has also been reported from lomustine administration, particularly at 3-weekly dosing intervals (49, 77). Thus, a 4–6-week dosing interval is recommended.

Outcomes with lomustine at 30-90 mg/m^2^ orally every 3–6 weeks as the leading addition to palliative care have been described in five dogs with histologically confirmed intracranial glioma (MST, 39 days; range, 18–120) and 16 dogs with a presumed diagnosis (MST, 135 days; range 5–520) (6, 45, 47). Survival was measured from advanced imaging diagnosis and tumor location was supratentorial (hemispheric and/or diencephalic) for all patients.

Surgical resection and adjuvant oral lomustine at 60-80 mg/m^2^ every 4–8 weeks was added to palliative treatment in a total of six confirmed cases of intracranial glioma from three different studies (6, 47, 48). Median survival time from diagnosis in these cases was 165 days (range, 60–720), and one dog was alive at the time of study finalization, 630 days after diagnosis. All six tumors were hemispheric.

The outcomes of two high-grade oligodendrogliomas receiving hypo-fractionated radiotherapy with a linear accelerator and adjuvant lomustine have been reported independently (50, 51). In one dog, lomustine courses at 60 mg/m^2^ every 6–9 weeks were initiated 120 days after MRI diagnosis as a rescue due to progressive disease confirmed with MRI (50). Treatment intervals were increased to every 9–14 weeks after 1 year and the dog lived 910 days. In the second case, lomustine chemotherapy (60 mg/m^2^ every 3 weeks for the first two courses, then every 4 weeks) was initiated concomitantly with radiotherapy and the patient survived 356 days after imaging diagnosis (51). Both oligodendrogliomas were hemispheric. Another two dogs with presumptive intracranial glioma received stereotactic radiotherapy and adjuvant lomustine and they survived 388 and 658 days from diagnosis, respectively (52). The dog with the longest survival had an infratentorial tumor, whereas in the other case, the tumor was supratentorial. Finally, one case with confirmed astrocytoma in the right cerebral hemisphere-diencephalon underwent surgical debulking followed by conventional multi-fractionated radiotherapy and lomustine (60-80 mg/m^2^ every 4 weeks) and was euthanized after 90 days due to intractable seizures and hyperthermia (47).

#### 4.1.3. Carmustine

Carmustine (1,3-Bis(2-chloroethyl)-1-nitrosurea, BCNU) is a non-phase-specific alkylating agent that interferes with DNA and RNA synthesis and repair (74). Its use has been described in one dog with histologically confirmed glioma in the right cerebral hemisphere (57). A dose of 50 mg/m^2^ intravenously over 20 min was administered every 6 weeks, leading to resolution of the severe presenting clinical signs even after discontinuation of palliative corticosteroids. Mild neutropenia occurred at posttreatment days 5 to 7. Partial remission was confirmed at week 18 via computed tomography showing marked reduction in tumor size (~75%). At week 24, the dog’s neurological status started to gradually deteriorate and was eventually euthanized 213 days after imaging diagnosis.

#### 4.1.4. Hydroxyurea

Hydroxyurea is an antimetabolite that specifically affects the S stage of the cell cycle and has been used as a chemotherapeutic agent for the management of canine intracranial meningiomas at a dose of 30-50 mg/kg orally three times a week (78–80). The only potential side effects of hydroxyurea were methemoglobinemia in one dog and suspected cumulative thrombocytopenia in another out of 43 reported cases (78–80). One dog with suspected supratentorial glioma and treated with stereotactic radiotherapy has been reported to receive adjuvant hydroxyurea and to survive 484 days when it was lost to follow-up (52). Hydroxyurea plus imatinib, a selective inhibitor of tyrosine kinase (5-8 mg/kg orally once daily), and prednisolone were also administered in a dog with a confirmed astrocytoma in the pons (58). The patient survived 1,155 days from initiation of therapy after diagnosis, when it was euthanized due to neurological decline and MRI confirmed tumor progression.

#### 4.1.5. Toceranib phosphate

Toceranib phosphate is a multitargeted tyrosine kinase inhibitor which blocks a variety of receptors such as platelet derived growth factor receptor alpha and vascular endothelial growth factor receptor 2, reportedly overexpressed in canine gliomas (81–84). Treatment with toceranib has shown benefit in several cancers in dogs and doses ranging from 2.4–2.9 mg/kg three times weekly provide drug exposure sufficient for target inhibition while resulting in decreased adverse effects (gastrointestinal, neutropenia, lameness) than label doses (3.25 mg/kg three times weekly) (81, 82, 85). In canine gliomas, additionally to the two above-described cases receiving it as rescue therapy after surgical debulking and temozolomide chemotherapy (53), it has also been used as adjuvant treatment to stereotactic radiotherapy in another presumed supratentorial glioma that survived 257 days (52).

#### 4.1.6. Cytarabine

Cytarabine (cytosine arabinoside) is a compound isolated from a sea sponge (86). Cytarabine is metabolized to an active drug that inhibits DNA synthesis (87–89). It was once thought that its action was via inhibition of the enzyme DNA polymerase, but the exact mechanism of action might be more complex (87–89). The most common use of cytarabine is as part of the treatment of meningoencephalomyelitis of unknown origin (MUO), lymphoma, and myeloid neoplasms (90–92). Four cases with confirmed intracranial glioma have been documented to receive subcutaneous cytarabine 50 mg/m^2^ given four times over 2 days (200 mg/m^2^ total dose) every 3 weeks as their MRI lesions were found most consistent with MUO granuloma and confirmed as glioma postmortem (two oligodendrogliomas and two astrocytomas, all high-grade) (6). Their MST was 22 days (range, 2–84). Tumor location was hemispheric in two cases, and diencephalic and infratentorial, respectively, for the remaining two dogs.

### 4.2. Investigational therapies

The value of novel chemotherapeutic agents, either alone or combined with other treatments, or delivery of drugs in a more targeted manner to bypass the limitations of systemic treatments; is actively investigated in glioma-bearing dogs with the hope that results will eventually translate into efficient new therapeutic strategies that improve current outcomes in human gliomas.

#### 4.2.1. Intratumoral temozolomide

Further to the above reported regimens including oral temozolomide for the treatment of canine intracranial glioma patients, biopolymer microcylinders containing temozolomide (1 mg) and gadolinium (0.25 mg) were implanted into partially resected tumors of four dogs with supratentorial glioma (55). All dogs recovered well from the craniotomy and implantation procedure. One dog was euthanized nine days after discharge due to splenic hemangiosarcoma. The remaining three dogs were alive and neurologically normal at short-term follow-up, 1 month after the procedure. Further follow-up was provided for two of these cases, the first of them developed moderate and progressive pneumocephalus that required surgical treatment 3 months post-implantation and was well 4 months after initial therapy. The last case in this series had continued experiencing seizures since the initial treatment and showed substantial tumor regrowth on one-month follow-up MRI. Neurological status had continued deteriorating 2 weeks later at which stage the patient was withdrawn from the study and no further information on survival was provided.

Another study documented the use of stereotaxis-guided convection-enhanced delivery (CED) of polymeric magnetite nanoparticles encapsulating temozolomide (5 mg/kg) in 10 dogs with spontaneous supratentorial tumors (56). In seven dogs, the infusion accurately targeted the tumor mass as determined on immediate postoperative MRI. All included tumors were suspected to be gliomas based on MRI; however, histologic diagnosis was obtained post-mortem in four cases confirming three were high-grade astrocytomas whereas the remaining one was a cystic meningioma. Dogs with confirmed glioma had a MST of 38 days post-CED (range, 1–82) and MST in those with presumed glioma was 89 days (range, 44–722), though the patient with the longest survival was alive at the time of study completion. One dog died on postoperative day 1 from a likely herniation event as it had a very large tumor and severe preoperative clinical signs.

The above approaches were supported by evidence in murine models indicating that intratumoral delivery of temozolomide is significantly more effective in improving survival time than systemic temozolomide, while decreasing systemic toxicity (93).

#### 4.2.2. Convection-enhanced delivery of irinotecan hydrochloride

Irinotecan hydrochloride (camptothecin-II, CPT-11) is a water-soluble derivate of the potent alkaloid anticancer agent camptothecin and acts as a specific topoisomerase I inhibitor that might induce apoptosis, cell cycle arrest and senescence secondary to double-stranded DNA breaks (94). Its activity has been demonstrated *in vitro* and *in vivo* in dogs (59, 67). The reduction in viable populations observed in canine glioma cell lines suggested that a modest clinical response, similar to that in humans, could be expected in canine patients (67). Therefore, the use of stereotaxis-guided CED of liposomal nanoparticles containing irinotecan hydrochloride in nine dogs with supratentorial intracranial glioma, either hemispheric or diencephalic, was investigated (59). Maximum percentage decrease in MRI-measured tumor volume was 88% with five tumors having a decrease of 40% or greater, whereas the rest had durable static disease. Transient (4–5 days) post-procedural lethargy was reported by owners. A decreased neurological status was noted in two cases after the procedure, observed MRI abnormalities were consistent with brain edema and patients responded to corticosteroid therapy both clinically and radiologically. Areas of encephalomalacia were confirmed postmortem in the most severely affected dog. The MST from MRI diagnosis was 190 days (range 126–611). Two dogs were euthanized due to unrelated reasons (hemangiosarcoma and pancreatitis, respectively), two cases were still alive at the time of conclusion of the study (surviving 611 and 181 days, respectively, and with the latter showing 88% reduction in tumor volume), and the remaining dogs were euthanized due to tumor progression except for one case that died during status epilepticus.

#### 4.2.3. Convection-enhanced delivery of cetuximab

Cetuximab is a human-mouse chimeric monoclonal antibody that binds specifically to the epidermal growth factor receptor (EGFR, most common glioma mutation in humans), preventing dimerization and activation of the tyrosine kinase and thereby inhibiting downstream signal transduction (95–98). It has shown efficacy as a single agent in a series of advanced human cancers by reducing cell proliferation, inhibiting tumor angiogenesis, and enhancing radiosensitivity through promotion of radiation-induced apoptosis with inhibition of radiation-induced damage repair (99). Systemic administration of cetuximab has been evaluated in phase II clinical trials in human patients with recurrent high-grade glioma and demonstrated minimal toxicity but limited efficacy and survival benefit (100–102). Subsequently, *in vitro* treatment with cetuximab conjugated iron-oxide nanoparticles (cetuximab-IONPs) resulted in a significant antitumor effect, greater than with cetuximab alone, due to more efficient cellular targeting and uptake, EGFR signaling alterations, EGFR internalization, and apoptosis induction in EGFR-expressing glioma CSCs (103). A significant increase in survival after CED of cetuximab-IONPs in rodents with EGFR-expressing glioblastoma xenografts was also noted. In dogs, EGFR overexpression has been shown in all glioma types and grades (83). Consequently, a pilot study evaluated the safety and efficacy of cetuximab-IONPs CED following open biopsy or partial tumor resection for the treatment of eight dogs with supratentorial low-grade oligodendrogliomas (60). Only two dogs developed mild postoperative complications, which resolved with medical therapy. All dogs underwent a single CED treatment of the cetuximab-IONPs over 3 days and did not receive any further adjuvant treatments. Volumetric analysis by MRI showed a median reduction in postoperative tumor size of 54.9% at 4–6 weeks follow-up. Five dogs were euthanized due to recurrence of neurological signs other than seizures, two due to recurrent seizures, and one died in his sleep. Median survival time was measured from surgery and was 248 days (range, 103–903).

#### 4.2.4. Targeted doxorubicin delivery via minicells

A recent study reported the use of anucleate, bacterially-derived minicells to deliver the chemotherapeutic doxorubicin (adriamycin) via EGFR targeting in 17 dogs with brain tumors (62). These included eight gliomas, two infratentorial and six supratentorial, of which, two could not be histologically confirmed as lesions were completely resolved at examination post-mortem. The anthracycline doxorubicin is a potent chemotherapeutic agent that shows activity against many different types of cancers including human and canine glioma cell lines; however, penetration into the brain is markedly limited by the BBB even when its integrity is disrupted by the invasion of tumor cells in brain tissue (62, 104–106). Doxorubicin is known to bind to DNA-associated enzymes, intercalate with DNA base pairs, and target multiple molecular targets to produce a range of cytotoxic effects (107). Systemic administration of doxorubicin might cause several adverse effects in dogs including myelosuppression, alopecia, gastroenteritis, stomatitis, and acute or cumulative cardiac toxicity (108). Additionally, high neurotoxicity has been documented when administered in association with osmotic BBB opening (109). In mouse xenograft models, EGFR targeted, doxorubicin loaded minicells (EGFR-minicells-dox) enabling intracellular drug release, eliminated the toxic side effects seen with systemic administration (110). Similarly, no adverse clinical, hematological, biochemical or neuropathological effects were observed with weekly intravenous injection of EGFR-minicells-dox in dogs with brain tumors (62). Minicells rapidly localized to the core of brain tumors and complete or marked tumor regression (>90% reduction in MRI-assessed tumor volume) were observed in 23% of the dogs, with more than 2 years remission in three cases. Palliative treatment was administered concomitantly, and MST was 264 days (range, 49–973) for all brain tumors. Survival specific to intracranial glioma cases included in this study is unavailable as individual case or tumor type outcome data were not provided.

#### 4.2.5. Metronomic chlorambucil

Chlorambucil is a water-soluble nitrogen mustard and an alkylating agent that minimally penetrates the normal BBB, with brain concentrations of 2% of plasma concentrations (111). Metronomic dosing has shown favorable results in some canine malignancies (112, 113). The mechanism of action might be antiangiogenic and immunomodulatory rather than cytotoxic as the metronomic dose might be too low to induce such effect. On this basis, a phase I canine clinical trial evaluated metronomic chlorambucil for intracranial glioma in addition to surgical resection (61). Ten cases were originally included based on MRI provisional diagnosis of hemispheric glioma; however, two dogs were subsequently removed from the study as histologic diagnosis was not glioma. Chlorambucil was administered orally at 4 mg/m^2^ every 24 h for at least 3 days before surgery and continued postoperatively until death or subclinical thrombocytopenia three dogs developed after 8–12 months of treatment, the only dose-limiting adverse event reported. Dogs additionally received lomustine postoperatively (60 mg/m^2^ every 4 weeks for 5 months). Previously prescribed prednisone was tapered over 1–2 weeks, and antiepileptic drugs were continued. Median surgical glioma specimen chlorambucil concentration was 37% (range, 0–178%) of serum concentration, indicating substantial but variable alteration of the BBB within canine gliomas. This might allow some effect of chlorambucil in certain cases as in a contemporary study, metronomic chlorambucil in brain tumor-bearing dogs failed to achieve plasma concentrations high enough to cause direct cytotoxic or growth inhibitory effects observed on human glioma cells *in vitro* (114). No myoclonus or increase in seizure activity, reported signs of chlorambucil neurotoxicity (115, 116), were noted. Six dogs had prolonged seizure-free intervals compared to prior to treatment initiation. Overall MST from surgery for all cancer-related deaths was 257 days (range, 64–860). Three dogs included in the survival analysis did not have a cancer-related death and were censored at days 5 (died of a brainstem ischemic infarct), 308 (completed radiotherapy for recurrence), and 860 (in complete remission).

#### 4.2.6. Clomipramine ongoing clinical trial

Finally, a recent case series included four dogs with confirmed hemispheric glioma enrolled in an ongoing clinical trial testing oral clomipramine at 1-2 mg/kg every 12 h in addition to corticosteroids and antiepileptic drugs (6). Their MST from MRI diagnosis was 236 days (range, 45–1,104) and cases included one high-grade tumor of each type, astrocytoma, oligodendroglioma, and undefined glioma, and a low-grade astrocytoma which survived the longest. No toxicities were noted. Clomipramine is a tricyclic antidepressant that was initially found to selectively kill neoplastic glial cells *in vitro* while sparing normal brain cells (117). The mechanism for this appears to be through mitochondrial targeted apoptosis via increase in caspase-3 activity (118).

## 5. Discussion

### 5.1. Chemotherapy responses in canine intracranial glioma: current state of knowledge

This review was undertaken to summarize the current state of knowledge regarding outcome after chemotherapy in intracranial glioma in dogs. Although there are several reports on the use of different chemotherapeutics, most data pertain to small case series and retrospective analyses, which carry inherent sources of bias (119). The lack of robustly designed randomized prospective clinical trials comparing various treatment options for intracranial glioma makes giving advice to clients regarding treatment decisions difficult.

In human medicine, the current first-line standard-of-care for intracranial high-grade gliomas consists of a multimodal approach including maximal safe surgical resection followed by radiotherapy and six cycles of temozolomide chemotherapy (76, 120). In dogs, a standard treatment regimen for intracranial glioma is lacking and controversy remains around the most appropriate approach which is also often conditioned by treatment modality availability and owners’ decisions. Recent evidence supports that surgical debulking, radiotherapy, and chemotherapy, either alone or in any combination, provide a significant survival benefit over palliative therapy (6). This was subsequently challenged, and assertions made that radiotherapy is superior to surgery in the treatment of canine intracranial gliomas and that chemotherapy is not an acceptable option for such purpose (13). These referred to a systematic review on brain tumor treatment in dogs that failed to show a clear difference in outcome between radiotherapy and surgery and that did not support lomustine as an effective treatment (15). However, the value of those observations was limited by the available data.

Although the body of knowledge on brain tumor treatment and outcomes in dogs has increased since then, there is still insufficient data on treatment efficacy for intracranial gliomas. From the available information we have this far, it seems that MST from imaging diagnosis in dogs with confirmed intracranial glioma receiving palliative care alone is just under 1 month (6, 45). Outcome data for histologically confirmed intracranial glioma after surgical resection as sole anticancer therapy (in addition to palliative care) is available in just 15 dogs (48, 121). The MST of 14 supratentorial cases included in one study with no control group was 66 days (range, 10–730 days) post-surgery (121), whereas the remaining case survived 60 days post-diagnosis (48). This compares favorably to palliative care; however, dogs in the case series that died within 7 days of surgery from complications related to the procedure were excluded, biasing survival analysis by their exclusion (121). Additional potential sources of bias in studies evaluating surgery as part of the treatment for canine intracranial glioma include the requirement for solitary and accessible masses, exclusion of tumors in the brainstem because of the likely higher mortality rate following surgery on that location, and exclusion of cases with severe neurologic dysfunction (122).

In humans, E.C. Holland evaluated survival data for patients diagnosed with glioblastoma and observed increasing survival times when comparing surgical biopsy only to extensive resection and, although to a lesser degree, when comparing extensive resection to extensive resection followed by radiation therapy, and the latter to ≥95% MRI-measured resection followed by both radiation and chemotherapy (26). Although there were essentially no long-term survivors, removal of tumor mass clearly increased longevity and quality of life. Nevertheless, glioma topographically diffuse nature and invasive behavior results in the inability to completely resect these tumors.

There is a lack of outcome information for dogs with confirmed intracranial glioma receiving radiotherapy as single anticancer modality. Median survival times for presumed cases consisted of 512 and 698 days from linear accelerator hypo-fractionated radiotherapy initiation in 16 and 38 dogs, respectively (123, 124), and 383 days from treatment course completion in 22 dogs undergoing hypo-fractionated stereotactic radiotherapy (46). These compare favorably to reported MSTs for suspected intracranial gliomas treated palliatively, 94 days in the control group of the latter study (46), and 37 days in another series (45). However, completion of the radiotherapy course without need of a second radiation protocol or other rescue treatments was a requirement for inclusion (46, 123, 124), which could have omitted an unknown number of cases unsuccessfully treated, and consequently, artificially inflated the reported survival times. Despite these methodology limitations and the lack of confirmed diagnoses, radiotherapy might still be effective; however, it is not always geographically or financially accessible to all owners.

Out of 127 dogs receiving chemotherapy for the treatment of intracranial glioma reviewed here, 32 clinical patients had lomustine (16 confirmed and 16 presumed gliomas), and 37 clinical patients (16 confirmed and 21 suspected) plus another 13 experimental patients (7 confirmed and 6 presumed), totaling 50 cases, were treated with temozolomide (6, 45–48, 50–53, 55, 56). Thus, as in human medicine, these two compounds are the most used chemotherapeutic agents for glioma in dogs (18, 19, 75, 76).

Survival times for cases in the literature solely receiving chemotherapy as anticancer treatment modality were longer than MST for those treated palliatively. The exception to this was treatment with cytarabine in four cases suspected to have MUO granuloma based on MRI (6). This could indicate that cytarabine is not an appropriate chemotherapy for gliomas. Nevertheless, it has recently been questioned whether the dosing and route of cytarabine administration the reported dogs received is optimal even for MUO (91).

When comparing reported histologically confirmed cases, outcomes following lomustine treatment alone did not differ much from palliative care (6, 45, 47); however, case numbers are very low. Also, the outcome of temozolomide as sole anticancer therapy has only been described in a single case (6). In presumed cases, treatment with lomustine alone yielded an over three-fold increase in MST compared to the palliative care control group of the same study, though dogs were not randomly assigned to different therapies (45). Other reported systemic chemotherapeutic regimens such as intravenous carmustine, oral hydroxyurea combined with imatinib, or oral clomipramine, have also anecdotally resulted in considerably longer survival times than palliative care (6, 57, 58).

Although only described in a few small case series utilizing different single or multiple chemotherapeutic agents, the combination of surgical resection with adjunctive chemotherapy in dogs with confirmed intracranial glioma also seems to prolong MST when compared to palliative treatment or surgical resection alone (6, 47, 48, 53, 60, 61, 121). On the other hand, a recent study failed to find a significant survival advantage in dogs irradiated for presumed glial tumors in combination with temozolomide (46). However, in another series evaluating stereotactic radiotherapy for presumptive intracranial gliomas, five dogs treated with one course of hypo-fractionated radiation also received chemotherapy with different compounds and had a MST of >658 days, a significantly longer survival than in those who did not receive adjuvant chemotherapy (MST, 230 days; *n* = 9) (52). Finally, only one dog has been reported to receive surgical resection followed by adjuvant radiotherapy and chemotherapy (lomustine) in the literature, surviving 90 days (47). Based on these contrasting observations and limited available data, it is currently not possible to assess whether multimodal therapy provides improved outcomes in dogs.

Anatomic tumor location could have influenced outcomes reviewed herein. A previous study on survival after hospital discharge in 51 dogs with palliatively treated primary brain tumors, including 14 gliomas, found that infratentorial tumors had a statistically significant poorer prognosis (125). However, a subsequent survival analysis in a larger cohort of intracranial gliomas following a variety of treatments found no associations between survival and tumor location (6). Despite limitations of available data, survival times reviewed here seem comparable in terms of lesion location as most of the cases had supratentorial gliomas (*n* = 122). Furthermore, with a MST of 438 days (range, 3–1,155), the five cases with infratentorial tumors did not appear to have a particularly poorer prognosis. It has also been hypothesized that tumor size could affect long-term survival. However, tumor volume determined via MRI was neither associated with nor predictive of outcome following surgery and adjuvant immunotherapy in a recent study including 47 dogs with histologically confirmed intracranial glioma (122).

Despite the weak evidence to support their efficacy in the treatment of canine intracranial glioma, it seems like most reported chemotherapeutics, either alone or combined with other anticancer therapies, could have some beneficial effect on survival. This far, no single chemotherapeutic agent appears advantageous over others, as evidenced by the observed survival periods of similar magnitude and with extremely wide ranges, and adverse effects of agents used do not seem to be limiting at the doses described above. However, interpretation of observations in the present literature must be cautious as further evidence is desperately needed so strong overall conclusions about the actual survival advantage provided by different chemotherapeutic compounds, or other therapies and their combinations, can be drawn.

### 5.2. Spontaneous animal model for human glioma

Dogs with glioma pose an attractive model for validation of novel therapies for the human counterpart (11). In addition to the phylogenetic proximity between human and dog compared to the mouse counterpart and the morphologic resemblances of gliomas between species, it might not be possible to establish the ideal standard-of-care for canine gliomas as this might not always be available to all institutions and a choice of or affordable to all owners. This lack of a treatment regimen deemed as “gold standard” allows for preclinical drug screening and clinical trials in this naturally occurring model of human glioma.

Noticeably, most reports on experimental therapies including those reviewed here are small case studies and analyze various histologic tumor types together. The latter could represent a major obstacle for translational research as therapeutic responses might differ between dogs and humans due to differences in histologic tumor subtype distribution among species (5, 6, 8, 17). Thus, stratification of responses by canine glioma subtype is of critical relevance for translational studies.

Large placebo control blinded clinical trials would also be necessary to further understand the role of investigational therapies in canine intracranial gliomas. Unusual results are more common when small populations are analyzed and are difficult to interpret without a randomly allocated contemporary control group. The small case numbers included in many reports imply greater probability for more extreme results and so the reported differences between outcomes in different study protocols are likely to represent random variation rather than true differences in effects of different treatment regimens.

Unfortunately, it is unlikely that placebo control blinded or randomized clinical trials comparing treatments will be carried out in dogs soon because of ethical and financial limitations in veterinary research. Thus, although imperfect, systematic review of previously published literature provides an alternative means of answering questions regarding relative efficacy of competing therapies. To carry out systematic review followed by meta-analysis, data collection and reporting must be consistent, and information available for extraction from published studies so statistical analysis is feasible. That includes documentation of responses to conventional therapies so meaningful statements regarding the actual survival advantage provided by novel therapeutic approaches can be made and inform of the potential benefit in humans.

### 5.3. Data collection and reporting

Current observations on therapeutic responses in canine intracranial gliomas might be anecdotal due to case numbers, possible publication bias, the abovementioned absence of control or placebo groups and lack of randomization, and other limiting factors including retrospective nature of studies, treatment modalities heterogeneity, lack of definitive diagnosis, and differences in inclusion and exclusion criteria as well as in definition of survival. Many of these prevent systematic review and subsequent meta-analysis of available data at this stage. Also, the use of survival as the sole outcome measure in most instances could prevent detection of more subtle treatment benefits. Ultimately, there is a need for standardized design and reporting of outcomes of treatment for intracranial gliomas in dogs, including chemotherapy.

#### 5.3.1. Diagnosis and classification of tumors

Where data was available for comparison, same treatment modalities yielded substantially increased MSTs for cases presumed of having intracranial glioma than for those histologically confirmed (Table 1) (6, 45, 47, 56). Eliminating misclassification of non-glial tumors by requiring histologic confirmation may introduce selection bias if the philosophical beliefs, financial availability, or other factors of owners, or clinical features and outcomes of dogs with intracranial glioma that are subjected to necropsy or surgical biopsy differ from those who do not. However, reported sensitivity for MRI diagnosis of neoplastic brain disease in dogs is 89% and specificity and sensitivity for the diagnosis of glioma are 93.7 and 84.4%, respectively (41, 126). This means other tumor types or intraaxial brain lesions such as cerebrovascular accidents or granulomas can be misdiagnosed as gliomas (4, 41, 56, 61, 127, 128). Potential inclusion of these in studies lacking histologic confirmation could have biased results toward more favorable outcomes. This observation confirms that insistence on a minimum of histologic diagnosis for publication remains critical for assessment of therapies.

Likewise, conventional MRI features of canine intracranial glioma type and grade overlap considerably resulting in low interobserver agreement, sensitivity, and specificity (6, 42, 43). Although advanced imaging analysis techniques could increase accuracy of glioma subtype diagnosis (129), a clear superiority for differentiation from other lesions such as MUO has not been shown yet (44).

Another limitation for assessment of therapies and the influence of tumor subtype in dogs is the fact that necropsy samples might not be representative of the original tumor phenotype, which could change with tumor progression or be influenced by treatments administered (53, 59). Thus, biopsies should ideally be obtained to confirm diagnosis prior to treatment, though they might also fail sometimes to reflect the overall histologic features of the glioma sampled (59), hampering accurate classification.

Finally, tumor typing and grading might vary between pathologists which could represent a potential source of bias. The Comparative Brain Tumor Consortium (CBTC) recently proposed a revised diagnostic classification of canine gliomas (5). The inter-pathologist agreement utilizing this schema is moderate, similar to pathologist agreement in human glioma studies (5, 130). Therefore, efforts should be made to use the CBTC classification when evaluating treatment responses in different glioma subtypes, so correlations between morphologic diagnosis and tumor behavior can be assessed.

#### 5.3.2. Definition of outcome measures

Reliable outcome measures are a basic requirement for treatments efficacy assessment and comparison. For brain tumors, the most reported endpoint has been overall survival. Albeit important, it might not be the most accurate measure of treatment efficacy in its own.

Firstly, there is a need to standardize survival time definitions to compare treatment outcomes. Most reports reviewed here measured survival from imaging diagnosis (6, 45, 47, 48, 50–53, 57–59), some measured it from specific anticancer treatment implementation (55, 56, 60, 61), and in a study combining irradiation with temozolomide, survival time was calculated from the end of radiation therapy (46). Specific anticancer treatment regimens are unusually implemented from diagnosis, even in cases solely receiving chemotherapy. Conversely, palliative care is usually started immediately in dogs with intention-to-treat and administered for a variable period before implementation of specific therapies. While this could bias selection by longevity, it does account for the add on effect of specific therapies in those patients, allowing for more accurate comparison to just palliative treatment. Therefore, survival from diagnosis might better reflect intention-to-treat in the absence of randomized prospective trials. In cases receiving specific therapies, it might also be useful to calculate survival time from treatment implementation to account for delays starting these (e.g., financial or geographic constraints).

In the studies reviewed here, patients were most commonly euthanized due to neurological status decline and/or intractable seizures. However, owner decisions to euthanize were not always synonymous of cancer progression-related death (61). Also, several dogs were euthanized for unrelated conditions (55, 59, 61). Careful annotation of these is necessary for documentation of disease-specific survival. Both, overall and disease-specific survival, are relevant outcome measures. From the owners’ perspective, overall survival data would be preferable because they do not always know whether there are other lesions in their dog that might contribute to the animal’s death within a specified period. On the other hand, veterinarians likely are more interested in disease-specific survival because they want to determine which treatment to recommend for an individual dog. Nevertheless, without consistency in data reporting, direct comparison is impossible.

Progression-free survival could contribute to more reliable treatment efficacy comparisons by circumventing the limitations of overall survival assessment due to owner decisions to euthanize. This was reported in some of the reviewed studies, where progression was defined as relapse or increase of neurological signs, or an increasing tumor size on advanced imaging (53, 61). Again, standardization of criteria is necessary so studies’ results are comparable. Response to treatment assessment should rely on simplified scoring systems comparing neurologic status to capture clinically relevant worsening of disease and then using that score to declare deterioration and progressive disease. Regular clinical evaluation results might be incorporated into MRI-based therapeutic response criteria (131, 132), but care must be taken not to incur euthanasia due to financial exhaustion (133).

#### 5.3.3. Exclusion of cases

Most of the reviewed publications documented the long-term survival times for the therapies they described. However, censored individuals (lost to follow-up, still alive at the time of analysis, died of non-tumor-related causes) were removed from the final survival analysis in several studies (45, 52, 53, 61). Herein, MST among reports of the same treatment modality with the same survival definition was calculated including those cases when the raw dataset was available. Although not randomized, inclusion of cases censored or excluded due to morbidity, mortality, and withdrawal from treatment before completion is meant to allow the reader to draw more accurate conclusions regarding the effectiveness of therapeutic interventions (15).

Of note is that in a recent multicentric survival analysis including 88 histologically confirmed canine intracranial gliomas, four were euthanized on presentation and 41 were euthanized upon diagnosis, all in view of the likely poor prognosis, severity of the clinical status of some dogs, or due to financial constraints (6). Thus, just over 50% of dogs with intracranial glioma were euthanized prior to receiving any treatment and excluded from further analysis to remove cases without intention-to-treat. Removing patients euthanized at diagnosis from analysis might play a role on the reported MSTs for dogs with intracranial glioma, as we might be inadvertently selecting cases for longevity. However, owner decisions to euthanize rather than natural death is a common limitation to analysis of glioma behavior in the light of treatment in veterinary medicine. These are not always based on clinical status severity, making it impossible to estimate the proportion of animals that were unaccounted for in the final analysis of each treatment modality that could have been pursued should their owners’ decisions had been different. In turn, lack of better definition of conventional treatments efficacy, makes it difficult for owners to opt for a specific treatment, a combination, or euthanasia.

## 6. Conclusion

Formal comparisons of treatment efficacy for intracranial gliomas in dogs are unavailable. Currently, the most documented chemotherapeutic approaches are oral temozolomide or oral lomustine which, although evidence is weak, seem to have some positive effect on survival compared to palliative treatment. However, this is the case for most of the chemotherapy compounds reviewed here. Thus, there is a need for standardized design of studies and reporting of outcomes so it is possible to conclude the effect of chemotherapy in canine intracranial gliomas and which agents are more efficacious more reliably.

So far evidence does not seem to support intratumoral delivery of temozolomide is more effective than oral administration although data is very limited. Convection-enhanced or other methods of intratumoral delivery of chemotherapy present some advantages over systemic administration; however, there is an inherent technical demand, requirement for intra-operative or immediately postoperative imaging and, overall, decreased availability.

Novel approaches including targeted delivery of drugs and new chemotherapeutic agents need further investigation. In the interim, oral temozolomide or lomustine are two chemotherapeutic agents readily available in the clinical setting that rarely cause limiting adverse effects. Therefore, their incorporation to the treatment of canine intracranial gliomas more routinely and documentation of the subsequent outcomes could provide the necessary evidence to support their standard use in these tumors. This, in turn, would provide information for comparison with the effect of novel chemotherapeutic approaches, allowing to assess more accurately their potential benefit over standardly used chemotherapeutic agents in human glioma and increasing the chances of translation of results in human therapeutic trials. Also, it would allow to assess combinations of temozolomide or lomustine with other modalities such as surgery and/or radiotherapy and, in the absence of randomization and control groups, to better establish whether canine intracranial glioma might benefit from multimodal approaches like the human counterpart.

Although ideal, it is unlikely that a randomized trial to compare different modalities across all types of gliomas, or even for selected subtypes, will be carried out unless the funding environment for veterinary medicine changes. Therefore, it would be helpful if sufficient detail were included to permit independent analysis of the published data or at least for such data to be available as supplementary information when published. Also, creation of a mutually accessible international database introducing the suggested standardized information could enable evidenced identification of the best treatments for intracranial gliomas in dogs, including the most effective chemotherapeutic agent, and definition of optimal recommendations for owners.

## Author contributions

RJ-L: Conceptualization, Data curation, Investigation, Methodology, Validation, Visualization, Writing – original draft, Writing – review & editing, Formal analysis, Funding acquisition.

## Abbreviations

BBB: blood–brain barrier
CBTC: Comparative Brain Tumor Consortium
CED: convection-enhanced delivery
cetuximab-IONPs: cetuximab conjugated iron-oxide nanoparticles
CNS: central nervous system
CSCs: cancer stem-like cells
EGFR: epidermal growth factor receptor
EGFR-minicells-dox: epidermal growth factor receptor targeted doxorubicin loaded minicells
MRI: magnetic resonance imaging
MST: median survival time
MUO: meningoencephalitis of unknown origin
